# Identification of *Lonepinella* sp. in Koala Bite Wound Infections, Queensland, Australia

**DOI:** 10.3201/eid2501.171359

**Published:** 2019-01

**Authors:** Holly Angela Sinclair, Paul Chapman, Lida Omaleki, Haakon Bergh, Conny Turni, Patrick Blackall, Lindsey Papacostas, Phillip Braslins, David Sowden, Graeme R. Nimmo

**Affiliations:** Pathology Queensland, Herston, Queensland, Australia (H.A. Sinclair, H. Bergh, G.R. Nimmo);; Caboolture Hospital, Caboolture, Queensland, Australia (P. Chapman);; Queensland Institute of Medical Research Berghofer, Herston (P. Chapman);; The University of Queensland, Brisbane, Queensland, Australia (L. Omaleki, C. Turni, P. Blackall);; Sunshine Coast University Hospital, Birtinya, Queensland, Australia (L. Papacostas, D. Sowden);; University of New England, Armidale, New South Wales, Australia (P. Braslins);; Griffith University, Gold Coast, Queensland, Australia (G.R. Nimmo)

**Keywords:** *Lonepinella*, koala bite, wound infection, *Phascolarctos cinereus*, *Pasteurellaceae*, *Lonepinella koalarum*, saliva, bacteria, zoonoses, 16S rRNA, *rpoB*, *infB*, *recN*, colony morphology, housekeeping genes, Australia

## Abstract

We report 3 cases of koala bite wound infection with *Lonepinella koalarum*–like bacteria requiring antimicrobial and surgical management. The pathogens could not be identified by standard tests. Phylogenetic analysis of 16S rRNA and housekeeping genes identified the genus. Clinicians should isolate bacteria and determine antimicrobial susceptibilities when managing these infections.

*Lonepinella koalarum*, a species present in koala (*Phascolarctos cinereus*) feces, is a gram-negative bacterium that can degrade tannin protein complexes (*1*). This bacterium is the only species of the genus *Lonepinella,* a member of the family *Pasteurellaceae*. *L. koalarum–*related strains have been identified in koala gingiva (*2*). We report 3 cases of human wound infection involving *Lonepinella*-like organisms occurring after koala bites in Queensland, Australia.

## The Study

In 2014, case-patient 1, a 69-year-old female wildlife worker from the Sunshine Coast region of Queensland, Australia, sought treatment for left wrist puncture wounds and a 2-cm laceration to the dorsum of her left hand after a koala bite. The wound was cleaned, and oral amoxicillin/clavulanic acid was administered. Increased erythema and edema developed after 48 hours. Surgical debridement was required, and intravenous piperacillin/tazobactam was given for 6 days, followed by oral trimethoprim and sulfamethoxazole; full recovery was achieved. A Gram stain revealed gram-positive and -negative organisms. After 48 hours of culturing, we identified *Staphylococcus sciuri* and 2 gram-negative coccobacilli (MS14434 and MS14435).

After this case, we reviewed records and found a similar previous incident. In 2012, case-patient 2, a 62-year-old male wildlife worker from Toowoomba, Queensland, Australia, went to a general practitioner for treatment of a koala crush-bite injury to the thumb. After increased pain, swelling, fever, and malaise developed, he sought hospital care. He had an open wound (2-mm long, 5–8-mm wide, 20-mm deep) with purulent discharge. Surgical debridement was required, and intravenous ticarcillin/clavulanic acid was administered for 4 days, followed by oral amoxicillin/clavulanic acid for 7 days; clinical improvement occurred. We cultured specimens obtained during the operation and found *Fusobacterium nucleatum*, *Staphylococcus aureus*, and an unidentifiable gram-negative bacillus (MS14436).

In 2015, case-patient 3, a 66-year-old woman from Brisbane, Queensland, Australia, sought hospital treatment for a koala bite wound on her right hand. Surgical debridement and washout revealed pus within the thenar muscle compartment and metacarpophalangeal joint. Intravenous piperacillin/tazobactam was given, and the patient’s condition improved. We cultured swabs of specimens acquired before and during surgery and isolated an unidentified gram-negative bacillus (MS14437).

We cultured all isolates on chocolate agar in 5% CO_2_ for 48 h and recorded growth in different culture conditions (Table 1). We performed biochemical reactions, sugar utilization, and cultures using in-house methods and commercial identification products API 20NE Microbial Identification Kit (bioMérieux, https://www.biomerieux.com/), RapID NH System (Remel, http://www.remel.com/Clinical/Microbiology.aspx), RapID ANA II System (Remel), and VITEK 2 GN and NH ID cards (bioMérieux). We performed matrix-assisted laser desorption/ionization time-of-flight mass spectrometry using the VITEK MS IVD database (bioMérieux) and performed antimicrobial susceptibility tests per the European Committee on Antimicrobial Susceptibility Testing (EUCAST) guidelines (http://www.eucast.org/clinical_breakpoints/) for *Pasteurella multocida* (*3*). We used Etest (bioMérieux) to determine MICs.

**Table 1:** Phenotypic characteristics of 4 clinical isolates obtained from koala bite wound infections, Queensland, Australia, and *Lonepinella koalarum* ACM 3666

Growth characteristic or condition	MS14434	MS14435	MS14436	MS14437	ACM 3666
Growth requirement					
X factor	–	–	–	–	–
V factor	–	–	–	–	–
Biochemical reaction					
Acetoin, Voges-Proskauer test	–	+	+	–	+
Arginine arylamidase	+	–	–	+	–
β-galactosidase	–	–	–	+	–
β-glucosidase	–	+	+	+	+
β-xylosidase	+	–	–	–	+
Catalase	–	–	–	–	–
H_2_S	–	–	–	–	–
Indole	–	–	–	–	–
Leucine arylamidase	+	+	+	+	+
Ornithine decarboxylase	–	–	–	–	–
Oxidase	+*	+*	+*	+*	+*
Phenylalanine arylaminidase	+	+	+	–	+
Urease	–	–	–	–	–
Courmarate	–	+	–	+	–
Maltotriose	+	+	+	+	+
N-acetyl-D-glucosamine	+	+	+	–	+
Phenylphosphonate	+	–	–	–	+
Phosphatase	+	+	+	+	+
Nitrate reduction	–	–	–	–	+
Hydrolyzed esculin	–	+	+	+	+
Sugar utilization					
Glucose	+	+	+	+	+
Sucrose	+	–	–	+	+
Lactose	–	–	–	–	–
Maltose	–	–	–	–	+
Mannose	+	+	+	+	+
Xylose	–	–	–	–	+*
Mannitol	–	–	–	–	–
Malate	+	+	+	+	+
D-cellobiose	–	–	–	+	+
Media type					
Horse blood agar	+	+	+	+	+
Chocolate agar	+	+	+	+	+
Bacitracin agar	+	+	+	+	+
Brain Heart yeast	+	+	+	+	+
MacConkey with crystal violet	–	–	–	–	–
Hemolysis on horse blood agar	–	–	–	–	–
Temperature and atmospheric conditions					
Room temperature	+	+	+	+	+
28°C Aerobic	+	+	+	+	+
35°C Aerobic	+	+	+	+	+
35°C in 5% CO_2_	+	+	+	+	+
35°C Microaerophilic	+	+	+	+	+
35°C Anaerobic	+	+	+	+	+
42°C Aerobic	–	–	–	+	–

*Weak positive.

We performed DNA amplification and sequencing of 16S rRNA (*4*), *rpoB* (*5*), *recN* (*6*), and *infB* (*7*) genes as previously published and deposited sequences in GenBank (Appendix Table 3). We used Geneious 10.0.6 (https://www.geneious.com) to align and analyze sequences. We performed a neighbor-joining analysis of 16S rRNA sequences by using Jukes-Cantor corrections and calculated bootstrap support in MEGA version 6 (https://www.megasoftware.net). We calculated the similarity matrix using MUSCLE (https://www.ebi.ac.uk/Tools/msa/muscle) and predicted genome relatedness using a previously published formula using the *recN* gene (*8*,*9*).

The colony morphology of the 2 isolates from case-patient 1 were distinctly different from each other; MS14434 was morphologically similar to MS14437, and MS14435 was similar to MS14436. Optimal colony growth was seen on chocolate agar with 5% CO_2_ and under microaerophilic conditions (Table 1). Results from commercial identification systems were mostly inconsistent (Appendix Table 1); note that *L. koalarum* is not included within the commercial databases used.

The MICs of all the antimicrobial drugs tested for MS14434 and MS14435 were low (Appendix Table 2); MICs for MS14436 and MS14437 were higher. MS14436 and MS14437 were resistant to benzylpenicillin when applying *P. multocida* breakpoints (*3*).

We compared 16S rRNA and *rpoB* gene sequences from our isolates with those available in public databases, including GenBank, but confident organism identification was not possible. The 16S rRNA phylogenetic analysis clustered all 4 isolates distantly from *L. koalarum* (ACM 3666), albeit within the same group (Figure).

**Figure:**
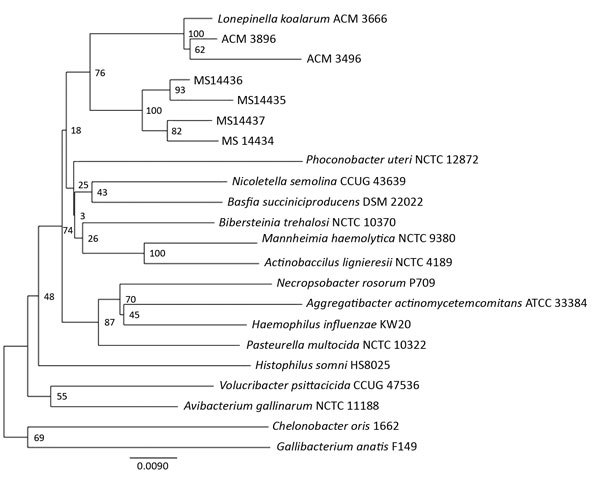
Neighbor-joining phylogenetic analysis of 16S rRNA gene sequences of 4 clinical isolates obtained from koala bite wound infections in 3 persons (MS14434–7), Queensland, Australia, 3 *Lonepinella koalarum* ACM isolates, and members of the *Pasteurellaceae* family. Scale bar represents nucleotide substitutions per site.

For the *rpoB* gene sequence, an identity of 85%–88% for genera and 95% for species has been suggested for *Pasteurellaceae* (*2*,*10*,*11*). All isolates of this study had an identity of >95% for the *rpoB* gene of ACM 3666 (Table 2), indicating a close genetic relationship with *L. koalarum*. A minimum level of 83%–85% identity of the partial *infB* gene has been shown between members of the *Pasteurellaceae* family at the genus level (*12*). The partial *infB* gene sequence of MS14434 had a high similarity (99.78%) to the corresponding sequence in ACM 3666, and the partial *infB* genes of the other 3 isolates are at the lower limit of the 83%–85% threshold. These 3 isolates also shared <85% similarity with the *recN* gene of ACM 3666 and >99% similarity with the *recN* gene of each other. The *recN* gene of MS14434 had 97.17% similarity with that of ACM 3666. Using the *recN* similarity index (*8*), we are 95% confident that these 3 isolates are a species other than *L. koalarum* within the genus *Lonepinella*, and MS14434 is most likely *L. koalarum*.

**Table 2:** Similarity matrix of 16S rRNA, *rpoB*, *infB*, and *recN* gene sequences of 4 clinical isolates obtained from koala bite wound infections, Queensland, Australia, and *Lonepinella koalarum* ACM 3666*

Gene and isolate	MS14434	MS14435	MS14436	MS14437	ACM 3666
16SrRNA					
MS14434	100				
MS14435	96.52	100			
MS14436	96.50	98.45	100		
MS14437	97.48	98.16	98.00	100	
ACM 3666	94.82	95.26	96.00	95.45	100
*rpoB*					
MS14434	100				
MS14435	95.77	100			
MS14436	96.54	97.69	100		
MS14437	95.96	99.42	97.88	100	
ACM 3666	95.96	95.96	95.00	96.35	100
*infB*					
MS14434	100				
MS14435	84.34	100			
MS14436	83.89	98.66	100		
MS14437	84.34	96.20	95.75	100	
ACM 3666	99.78	84.12	83.67	84.12	100
*recN*					
MS14434	100				
MS14435	83.90	100			
MS14436	84.13	99.06	100		
MS14437	83.97	99.53	99.37	100	
ACM 3666	97.17	83.97	84.21	84.05	100

*Values are percentage identity.

Each case manifested with purulent skin and soft tissue infection requiring surgical washout and debridement, similar to infections linked to *Pasteurella* in dog and cat bite wounds (*13*). MICs of amoxicillin and clavulanic acid, third-generation cephalosporins, and ciprofloxacin were low for all isolates. MIC determination should be sought because 2 isolates were nonsusceptible to benzylpenicillin on the basis of *P. multocida* interpretation criteria (*3*). For resolution of infection, surgical drainage might be required in addition to antimicrobial drug therapy.

In the original study of *L. koalarum* (*1*), 7 isolates were grouped into 4 biovars (a–d), and 16S rRNA sequencing demonstrated high similarity (97.6%–99.8%). A threshold of 93%–94% identity between 16S rRNA gene sequences has been described for differentiating members of *Pasteurellaceae* at the genus level (*5*) and >97% at the species level (*10*). Although all 4 isolates in our study showed >93% similarity to *L. koalarum* ACM 3666 in their 16S rRNA genes, none of them reached 97% similarity. Both *infB* and *recN* gene sequences indicated a close relationship between MS14434 and ACM 3666; however, 16S rRNA and *rpoB* gene sequences showed the same level of similarity between all 4 isolates and the reference *L. koalarum* strain. This disagreement between genes could be a result of lateral gene transfer; lateral gene transfer of housekeeping genes has been described as a reason for incongruence between 16S rRNA and housekeeping gene phylogeny (*14*).

## Conclusions

Clinical and microbiological suspicion is required when assessing bacteria from koala bite wounds. Phenotypic and biochemical colony characteristics are often unreliable at assigning isolates to a genus and species within the *Pasteurellaceae* family, and identification with commercial kits is not always possible. *Pasteurellaceae* spp., including *L. koalarum,* can be identified by using matrix-assisted laser desorption/ionization time-of-flight mass spectrometry with updated spectra (*15*). Clinical laboratory identification methods involve sequencing the 16S rRNA gene and searching nucleotide databases. As seen in this investigation, this approach can be inconclusive, and phylogenetic analysis of sequences including housekeeping genes might be required.

In summary, *Lonepinella* infections acquired after koala bites can cause clinically significant human skin and soft tissue disease. In this report, we identified possibly novel *Lonepinella*-like organisms with a combination of genetic analyses.

Appendix Identifications, MICs, and GenBank accession numbers of clinical isolates obtained from koala bite wound infections, Queensland, Australia.
