# Supplementary material for: Identification of Lonepinella sp. in Koala Bite Wound Infections, Queensland, Australia
Source: Emerg Infect Dis. 2019 Jan;25(1):153–6. doi: 10.3201/eid2501.171359 (PMC6302581; doi:10.3201/eid2501.171359)
Supplement: Appendix — Identifications, MICs, and GenBank accession numbers of clinical isolates obtained from koala bite wound infections, Queensland, Australia. [file 17-1359-Techapp-s1.pdf]

Article DOI: https://doi.org/10.3201/eid2501.171359

Identification of *Lonepinella* sp. in Koala Bite Wound Infections, Queensland, Australia

Appendix

Appendix Table 1. Suggested identifications for the 4 clinical isolates obtained from koala bite wound infections, Australia, and *Lonepinella koalarum* reference strain (ACM 3666) provided by commercial kits or systems

Identification method	MS14434	MS14435	MS14436	MS14437	ACM 3666
VITEK MS	*Haemophilus parainfluenzae*	*H. parainfluenzae*	No ID	*H. parainfluenzae* 99.8%	No ID
VITEK 2 GNI	*Sphingomonas paucimobilis* 97%	*S. paucimobilis* 99%	*S. paucimobilis* 98%	*S. paucimobilis* 99%	*S. paucimobilis 98%*
VITEK 2 NH	*Aggregatibacter segnis* 94%	*A. segnis* 89%	*A. segnis* 90%	*A. segnis* 87%	*Aggregatibacter aphrophilus 93%*
API 20 NE	*Mannheimia haemolytica* and *Bibersteinia trehalosi* 92.9%	*Brevundimonas vesicularis* 83.9%	*Chryseobacterium indologenes* 64.8%	*M. haemolytica* and *Pasteurella pneumotropica*	*M. haemolytica* and *B. trehalosi 94.2%*
RapID NH	*H. parainfluenzae*	*H. parainfluenzae*	*H. influenzae*	No ID	*H. parainfluenzae*
RapID ANA II	*Prevotella disiens*	No ID	No ID	No ID	*Capnocytophaga sp.*

ID, identification.

Appendix Table 2. Antimicrobial susceptibilities of 4 clinical isolates obtained from koala bite wound infections, Australia, and *Lonepinella koalarum* reference strain (ACM 3666)*

	MIC, mg/L					*Pasteurella multocida* EUCAST
Antimicrobial drug	MS14434	MS14435	MS14436	MS14437	ACM 3666	S breakpoint, mg/L
Benzylpenicillin	<0.002	<0.002	1.5	3	1	≤0.5
Ampicillin	0.75	0.75	1	1	0.5	≤1
Amoxicillin	<0.016	<0.016	0.75	ND	ND	≤1
Amoxicillin clavulanate	<0.016	<0.016	0.75	1	1	≤1
Piperacillin tazobactam	0.5	0.75	1.5	0.25	1	No breakpoint
Cefotaxime	0.012	0.016	0.023	0.023	0.016	≤0.03
Ciprofloxacin	<0.002	<0.002	0.016	0.012	0.016	≤0.06
Doxycycline	<0.016	<0.016	1	0.38	2	≤1
Trimethoprim/ sulfamethoxazole	0.002	0.006	0.006	0.002	0.032	≤0.25

*EUCAST, European Committee on Antimicrobial Susceptibility Testing; ND, not done; S, susceptibility.

Appendix Table 3. GenBank accession numbers for 16S rRNA, *rpoB*, *infB*, and *recN* gene sequences of 4 clinical isolates obtained from koala bite wound infections, Australia

Gene	Isolate	GenBank accession no.
16S rRNA	MS14434	MH745052
	MS14435	MH745053
	MS14436	MH745054
	MS14437	MH745055
rpoB	MS14434	MH744673
	MS14435	MH744674
	MS14436	MH744675
	MS14437	MH744676
infB	MS14434	MH744677
	MS14435	MH744678
	MS14436	MH744679
	MS14437	MH744680
recN	MS14434	MH744681
	MS14435	MH744682
	MS14436	MH744683
	MS14437	MH744684
